# Ethische Grenzentscheidungen in der Intensivmedizin

**DOI:** 10.1007/s00108-024-01781-5

**Published:** 2024-09-23

**Authors:** Benedikt Florian Scherr, Philipp Karl Buehler

**Affiliations:** 1 Zentrum für Intensivmedizin, Kantonsspital Winterthur, Brauerstrasse 15, 8401 Winterthur, Schweiz; 2 University of Zurich, Zürich, Schweiz

**Keywords:** Patientenwille, Entscheidungsfindung, Zeitlich begrenzter Therapieversuch, Nicht sinnhafte Therapie, Triage, Patient′s will, Decision-making, Time-limited trial, Treatment/futility, Triage

## Abstract

**Hintergrund:**

Ethische Grenzentscheidungen sind ein wesentlicher Bestandteil der Intensiv- und Notfallmedizin. In Akutsituationen müssen oft unter Zeitdruck und mit unvollständigen Informationen rasche Entscheidungen getroffen werden. Diese Entscheidungen werden durch Faktoren wie Ökonomisierung, Ressourcenmangel und zunehmende technische Möglichkeiten erschwert.

**Fragestellung:**

Welche Entscheidungshilfen und Faktoren können bei ethischen Grenzfällen in der Intensivmedizin herangezogen werden?

**Ergebnisse:**

Grundlegende ethische Prinzipien wie Patientenautonomie, Benefizienz, Nichtschaden und Gerechtigkeit bilden die Basis für medizinische Therapieentscheidungen. Die Evaluation des Patientenwillens durch Patientenverfügungen oder Stellvertreterkonsens ist entscheidend, wobei Patientenverfügungen oft unklar sind. Die Abschätzung der Lebensqualität gewinnt zunehmend an Bedeutung, wobei Instrumente wie die Clinical Frailty Scale (CFS) zur Anwendung kommen. Bei älteren Patienten sollte eine ganzheitliche Betrachtung erfolgen und nicht nur das chronologische Alter berücksichtigt werden. Bei Patienten mit fortgeschrittenen Grunderkrankungen ist ein multidisziplinärer Austausch besonders wichtig.

**Schlussfolgerung:**

Die Entscheidungsfindung in der Intensivmedizin erfordert eine sorgfältige Abwägung medizinischer, ethischer und individueller Faktoren. Trotz Fortschritten in der künstlichen Intelligenz und Prognosemodellen bleibt die menschliche Beurteilung unerlässlich. In Zeiten knapper Ressourcen sind ethisch vertretbare Triageprotokolle notwendig. Die Herausforderung besteht darin, diese Prinzipien und Faktoren in der klinischen Praxis anzuwenden und dabei die Individualität jedes Patienten zu berücksichtigen.

## Abstract (en)

**Background:**

Ethical decision-making is a cornerstone of intensive care and emergency medicine. In acute scenarios, clinicians often face rapid, high-stakes decisions concerning life and death, made more challenging by time constraints and incomplete information. These decisions are further complicated by economic constraints, limited resources, and evolving technological capabilities.

**Question:**

What decision-making aids and factors can be employed in ethical borderline cases within intensive care medicine?

**Results:**

Fundamental ethical principles such as patient autonomy, beneficence, non-maleficence, and justice form the basis for medical treatment decisions. Evaluating the patient’s will through advanced directives or proxy consensus is crucial, although advanced directives can be ambiguous. Assessing quality of life is increasingly important, with instruments such as the Clinical Frailty Scale (CFS) being utilized. For older patients, a holistic approach is recommended, focusing on overall health rather than chronological age. In patients with advanced underlying diseases, a multidisciplinary dialogue is essential.

**Discussion:**

Decision-making in intensive care medicine requires careful consideration of medical, ethical, and individual factors. Despite advances in artificial intelligence and prognostic models, human judgment remains crucial. During periods of resource scarcity, ethically sound triage protocols are required. The challenge lies in applying these principles and factors in clinical practice while respecting the individuality of each patient.

In der Intensiv- und Notfallmedizin müssen oft unter hohem Zeitdruck und mit unvollständigen Informationen lebenswichtige Entscheidungen getroffen werden [1]. Ethische Fragestellungen sind dabei zentral. In den letzten Jahren wurden diese Entscheidungen durch Ökonomisierung, Ressourcenmangel und technische Fortschritte immer komplexer, was häufig zu ethischen Konflikten und Dilemmata für das Behandlungsteam und die Patienten führt. Diese Grenzentscheidungen nehmen mit den heutigen intensivmedizinischen Möglichkeiten zu und stellen das medizinische Team vor große Herausforderungen. Die Frage nach der Lebensqualität im Kontext von Leidens- vs. Lebensverlängerung wird immer wichtiger. Dieses Thema gewinnt gesamtgesellschaftlich und ökonomisch an Bedeutung und wird uns in Anbetracht von begrenzten Ressourcen, Fachkräftemangel und demografischem Wandel künftig noch stärker fordern.

Seit vielen Jahren wird versucht, Therapieentscheidungen durch Abschätzung von Aufwand, Prognose und Outcome kritisch kranker Patienten mithilfe von Scores zu unterstützen, beispielsweise mit

- Acute Physiology And Chronic Health Evaluation (APACHE),
- Simplified Acute Physiology Score (SAPS) II und III,
- Sequential Organ Failure Assessment (SOFA),
- Therapeutic Intervention Scoring System (TISS) und
- Nine Equivalents of Nursing Manpower Use Score (NEMS).

Diese Systeme bleiben jedoch in ihrer Aussagekraft eingeschränkt. Ähnliches gilt für in den letzten Jahrzehnten entwickelte KI-Modelle, die Ergebnisse vorhersagen sollen, aber ebenfalls bisher nur einen begrenzten Nutzen haben. Ein Beispiel ist das Gradient-boosting-Maschinenmodell für die Mortalität im Krankenhaus [2, 3]. Dieses konnte für die 3‑ bis 14-Tage-Mortalität gute Ergebnisse zeigen [4]. Trotzdem findet es bisher im klinischen Alltag keinen Einsatz, da die Entscheidungsfindung mit Unterstützung durch moderne KI-Modelle eingeschränkt, komplex und multifaktoriell beeinflusst ist. Diese Modelle bieten zwar Einblicke und Prognosen, ersetzen jedoch nicht die ganzheitliche menschliche Betrachtung und individuelle Beurteilung jedes Patienten. Faktoren wie der klinische Zustand, die Prognose, die persönlichen Wünsche des Patienten und ethische Überlegungen spielen weiterhin eine entscheidende Rolle und müssen sorgfältig abgewogen werden.

Die Entscheidungsfindung auf der Intensivstation ist daher eine besondere Herausforderung für das Behandlungsteam. Sie erfordert eine sorgfältige Abwägung technischer Möglichkeiten sowie medizinischer, ethischer und individueller Patientenfaktoren.

KI-Modelle ersetzen nicht die ganzheitliche menschliche und individuelle Betrachtung jedes Patienten

Ziel des vorliegenden Beitrags ist es, Faktoren für ethische Grenzfälle zu erörtern, die die Entscheidungsfindung in der Medizin und Intensivmedizin beeinflussen können. Zudem soll der Beitrag dem klinischen Personal bei ethischen Grenzentscheidungen im Alltag Unterstützung bieten. Es werden allgemeine Grundsätze formuliert, keine speziellen Anweisungen. Diese Entscheidungshilfen und Statements entbinden nicht von der individuellen Verantwortung.

## Ethische Prinzipien, Patientenwille und Patientenverfügung

Grenzentscheidungen in der Intensivmedizin erfordern eine strukturierte und sorgfältige Vorgehensweise. Basis aller medizinischen Therapieentscheidungen sind folgende ethische Prinzipien, die in unserem täglichen Handeln eine zentrale Rolle einnehmen sollten [5].

- *Patientenautonomie*: Patienten haben das Recht, über ihre Gesundheitsversorgung zu entscheiden. Das behandelnde Team muss den Patienten alle relevanten Informationen für diese Entscheidungen zur Verfügung stellen und diese respektieren.
- *Fürsorge (Benefizienz)*: Ärzte sollen im besten Interesse der Patienten handeln und Maßnahmen ergreifen, die deren Wohl fördern. Dies kann im Widerspruch zur Autonomie stehen.
- *Nichtschaden (Nonmalefizienz)*: Ärzte sind verpflichtet, keinen Schaden zuzufügen. Behandlungen müssen sorgfältig abgewogen werden, um sicherzustellen, dass der Nutzen die Risiken überwiegt.
- *Gerechtigkeit*: Alle Patienten haben Anspruch auf faire und gleiche Behandlung, unabhängig von Faktoren wie Alter, Geschlecht, Rasse, Nationalität, Religionszugehörigkeit oder sozioökonomischem Status. Ressourcen sollen gerecht verteilt werden.

Es muss bei ethischen Grenzentscheidungen sichergestellt sein, dass alle Entscheidungen auf den ethischen Prinzipien der Patientenautonomie, Benefizienz, Nonmalefizienz und Gerechtigkeit basieren. Besondere Bedeutung erhält jedoch die Autonomie des Patienten durch Berücksichtigung von Patientenverfügungen und Vorsorgevollmachten. Eine Evaluation des Patientenwillens insbesondere in Grenzentscheidungen stellt das Team vor eine große Herausforderung. Jedoch ist zur Wahrung des ethischen Prinzips der Patientenautonomie der Patientenwille entscheidend. Bei urteilsunfähigen Patienten auf der Intensivstation kann dieser oft nicht direkt evaluiert werden. Dann bleibt die Patientenverfügung oder der Stellvertreterkonsens durch Angehörige, Lebenspartner oder gesetzliche Beistände als Alternative.

Viele Patientenverfügungen sind unklar oder enthalten mehrdeutige Informationen. Oft beschreiben sie nur aussichtslose Situationen, die bereits durch ethische Prinzipien abgedeckt sind. Zudem haben Patienten häufig falsche Vorstellungen von den Möglichkeiten und Limitationen der Intensivmedizin. Dies zeigte auch die Analyse von Fischer et al. [6], bei der zwei Drittel der Patienten davon ausgingen, unter mechanischer Beatmung sprechen zu können. Nur einer von 39 befragten Patienten zog die Möglichkeit einer Hirnschädigung im Rahmen von Reanimationsmaßnahmen in Erwägung. Trotz ihrer Unklarheiten und Einschränkungen bleiben Patientenverfügungen wichtig. Ihre besondere Bedeutung liegt im Ausfüllen und der damit verbundenen emotionalen Vorbereitung und Auseinandersetzung von Patienten und ihren Angehörigen mit möglichen gesundheitlichen Krisen sowie in der Formulierung des Patientenwillens für sich und ihre Angehörigen [7]. Fragen nach Wertevorstellungen des Patienten können in dieser Situation sehr helfen.

In einer Krisensituation können über die Evaluation einer Patientenverfügung auch der offene Dialog und Diskussionen mit Angehörigen begonnen werden, welche ebenfalls von besonderer Bedeutung sind. Dadurch kann das Behandlungsteam die zugrunde liegenden Werte, Interessen und bekannten Präferenzen des Patienten („substituted interests“) bestmöglich verstehen [8, 9]. Der direkt oder indirekt geäußerte Patientenwille sollte vom betreuenden Arzt in den Entscheidungsprozess einbezogen werden. Der mit zunehmender Betreuungszeit wachsende Informationsstand muss laufend in den Entscheidungsprozess einfließen [1].

In der Betreuung gesammelte Informationen müssen laufend in den Entscheidungsprozess einfließen

Ein temporäres Abweichen vom Patientenwillen kommt ausnahmsweise nur dann in Betracht, wenn sich nicht entscheiden lässt, ob der aktuell geäußerte Wille dem längerfristig zu vermutenden Patientenwillen entspricht. Eine solche Situation liegt etwa dann vor, wenn ein Patient nach einem Suizidversuch in intensivmedizinische Behandlung gelangt und bei Bewusstsein an seinem Todeswunsch festhält oder bei fehlendem Bewusstsein in einem schriftlichen Dokument oder durch entsprechende Zeugen seines Willens eine Behandlung ablehnt. Sein Wille muss aber im Verlauf der Behandlung reevaluiert werden [1]. Hier gilt primär die Notfallversorgung nach mutmaßlichem Willen.

## Sinnhaftigkeit der intensivmedizinischen Therapie

Ein weiterer Punkt der Entscheidungsfindung in einer ethischen Grenzsituation ist die Sinnhaftigkeit der intensivmedizinischen Therapie. Generell sollten intensivmedizinische Therapien sinnhaft sein. Eine Therapie gilt als sinnhaft, wenn

- sie eine medizinische Indikation hat und wahrscheinlich das Therapieziel erreicht und/oder
- die Durchführung dem Patientenwillen entspricht, ermittelt durch Aufklärung, Patientenverfügung oder Stellvertreterentscheidung.

Nichtsinnhafte Therapien werden mit „futility“ bezeichnet. „Futility“ bezieht sich auf das Fehlen einer medizinischen Indikation für eine Behandlung. Eine Maßnahme gilt als „futile“, wenn sie keine realistische Chance hat, das Behandlungsziel zu erreichen, und nur Belastungen verursacht. In solchen Fällen ist eine Therapiebegrenzung oder der Übergang zu einer palliativen Versorgung gerechtfertigt. Die individuellen Präferenzen und Werte des Patienten sind hierbei entscheidend [26]. Fehlt eine der Voraussetzungen oder ist das Therapieziel nicht mehr erreichbar, ist eine Therapiebegrenzung geboten. Diese kann den Verzicht auf zusätzliche Maßnahmen bzw. die Reduktion oder das Absetzen bestehender Maßnahmen umfassen. Die palliative Therapie muss zur Symptomkontrolle optimiert werden, insbesondere zur Linderung von Schmerzen, Luftnot, Übelkeit und psychosozialen Belastungen.

Die Sinnhaftigkeit von Intensivbehandlungen muss unter Berücksichtigung medizinischer Fakten und der Patientenpräferenzen sorgfältig abgewogen werden. Interdisziplinäre Entscheidungsfindung und offene Kommunikation sind entscheidend, wenn die besten Ergebnisse für den Patienten gewährleistet werden sollen [26]. Basis aller Therapien bleibt die Evaluation des Patientenwillens.

## Ethische Grenzentscheidungen und mögliche Einflussfaktoren

Neben den Basiskriterien zur ethischen Grenzentscheidung gibt es weitere Einflussfaktoren, welche die Therapieentscheidungen beeinflussen können. Untersuchungen zu intensivmedizinischen Therapieentscheidungen konnten hier einzelne Faktoren herausarbeiten. Hierzu gehören das fortgeschrittene Alter, die Lebensqualität, hämatoonkologische Grunderkrankungen, Polymorbidität oder limitierte Ressourcen, die einen Einfluss auf die Entscheidungsfindung insbesondere in Grenzsituationen bei Extracorporeal-life-support(ECLS)-Therapie haben können [13].

### Fortgeschrittenes Alter

Mit fortschreitendem Alter der Patienten wird der Einsatz intensivmedizinischer Maßnahmen oft zurückhaltender beurteilt. Bei geriatrischen Patienten werden Multimorbidität, abnehmende funktionelle Reserven und eingeschränkte Heilungsaussichten stärker gewichtet. Das chronologische Alter allein ist jedoch ein schlechtes Kriterium für Entscheidungen über intensivmedizinische Maßnahmen. Eine ganzheitliche multidisziplinäre Betrachtung, die kognitive Beeinträchtigungen, eingeschränkte Alltagsaktivitäten und Begleiterkrankungen einbezieht, ist erforderlich. In den letzten Jahren hat sich die standardisierte Evaluation der Clinical Frailty Scale (CFS) in der Intensivmedizin durchgesetzt; sie soll eine bessere Prognose in Bezug auf Komorbiditäten und die Aufnahmeursache ermöglichen und das Behandlungsteam in ihren Entscheidungen unterstützen [11, 12].

### Lebensqualität

Die kontinuierliche Weiterentwicklung medikamentöser und apparativer intensivmedizinischer Verfahren führt zu einer stetigen Zunahme an Behandlungsmöglichkeiten und ermöglicht heute Therapien und Lebensverlängerungen, die früher undenkbar waren. Therapiemöglichkeit und Lebensverlängerung bedeutet jedoch nicht zwangsläufig eine Verbesserung des Outcomes mit einer Zunahme der Lebensqualität. Daher rückt die maximal zu erreichende Lebensqualität zunehmend in den Fokus der Therapieziele.

Die maximal zu erreichende Lebensqualität rückt zunehmend in den Fokus der Therapieziele

Eine Prognose der Lebensqualität ist besonders in der Frühphase der intensivmedizinischen Versorgung schwierig. Seit einigen Jahren führen zunehmende Behandlungsmöglichkeiten häufig zur Durchführung lebenserhaltender Therapien, die im Verlauf zu ethischen Grenzentscheidungen und Dilemmata führen können. Ein Beispiel dafür ist der Einsatz des ECLS zur Überbrückung von Therapieentscheidungen, bekannt als „bridge to decision“.

Um die maximal zu erreichende Lebensqualität besser vorhersagen zu können, erfolgten in der Intensivmedizin zunehmend Outcomestudien, in denen die Lebensqualität nach invasiven intensivmedizinischen Maßnahmen mithilfe des Barthel-Index oder des Short-Form-Gesundheitsfragebogens SF-36 evaluiert wurde. Etabliert hat sich hier insbesondere bei geriatrischen Patienten die CFS. Durch Einsatz der CFS und unter Berücksichtigung der Grunderkrankung sind valide Aussagen zum erwartbaren Erholungspotenzial möglich, die mit den Angehörigen besprochen werden können [10].

### Palliative Grunderkrankung und/oder multiple Begleiterkrankungen

Hämatoonkologische Patienten sowie Patienten mit fortgeschrittenen pulmonalen, kardialen oder neurologischen Erkrankungen haben heutzutage nicht mehr per se eine schlechte Prognose und stellen eine große Herausforderung für die Intensivmedizin dar. Aufgrund neuer Therapieansätze erfolgt zunehmend eine individuelle Behandlung mit teils sehr guten Langzeitergebnissen. Hier ist der multidisziplinäre Austausch mit Hämatologen, Onkologen, Pneumologen und Kardiologen von besonderer Bedeutung. Angesichts der Komplexität der Erkrankungen und der wachsenden Behandlungsoptionen ist eine bestmögliche Versorgung intensivmedizinischer Patienten nur in interdisziplinären und interprofessionellen Teams möglich [13].

### Kommunikation

Neben den oben aufgeführten Einflussfaktoren kommt der Kommunikation mit den Angehörigen und dem Team eine besondere Bedeutung zu. Hier sollten ein multidisziplinärer Ansatz und eine offene Kommunikation mit den Bevollmächtigten gewählt werden. Dieser Ansatz ist essenziell für Entscheidungen über lebenserhaltende Maßnahmen in der Intensivmedizin. Laut Vig et al. [20] begründen Bevollmächtigte ihre Entscheidungen mit Gesprächen über Patientenpräferenzen, schriftlichen Dokumenten wie Patientenverfügungen, gemeinsamen Erfahrungen und persönlichen Werten, die in der Kommunikation berücksichtigt werden sollten. Ethiker und Kliniker bevorzugen hingegen oft die „substituted interests“ [8] in diesen Prozessen, um den mutmaßlichen Willen bestmöglich zu berücksichtigen. Mit dem Ziel, diese unterschiedlichen Wertemodelle zusammenzubringen, konnten folgende Ansätze aufgezeigt werden.

Nicoli et al. [21] beschreiben einen strukturierten Ansatz zur Entscheidungsfindung mit ihrem „Vier-Felder-Ansatz“. Dieser umfasst die medizinische Indikation, Patientenpräferenzen, Lebensqualität und den Kontext (familiäre, soziale, institutionelle, finanzielle und rechtliche Umfelder). Dieser Ansatz hilft, klinische Fälle systematisch zu analysieren und ethische Probleme zu klären, sodass der Patientenwille suffizient evaluiert werden kann.

Von besonderer Bedeutung sind die von Tulsky et al. [22] beschriebenen individuellen Informationsbedürfnisse am Lebensende und die Notwendigkeit verbesserter Kommunikation in kritischen Situationen. Eine gemeinsame Entscheidungsfindung umfasst drei Phasen:

- Informationsaustausch
- Beratung
- Entscheidungsfindung

Ärzte und Bevollmächtigte sollten vertrauensvoll zusammenarbeiten, um Entscheidungen im Sinne der Patientenpräferenzen treffen zu können. Dabei ist wichtig, dass der Informationsaustausch verständlich und in angemessener Umgebung erfolgt [23]. Schulungen, etwa zur Überbringung schlechter Nachrichten, sind dabei sehr nützlich und sollten Teil der ärztlichen Ausbildung sein.

## Unterschiedliche Therapiemöglichkeiten in Grenzentscheidungen auf der Intensivstation

In der Intensivmedizin gibt es bei Grenzentscheidungen verschiedene Therapieansätze, die je nach individuellen Patientenbedürfnissen und Prognosen gewählt werden. Einige der wichtigsten Therapiemöglichkeiten sind

- die kurative Intensivmedizin,
- die Palliativversorgung und Sterbebegleitung sowie
- der zeitlich begrenzte Therapieversuch („time-limited trial“ [TLT])

### Kurative Intensivmedizin

Bei der kurativen Intensivmedizin werden alle verfügbaren intensivmedizinischen Ressourcen und Techniken eingesetzt, um lebensbedrohliche Zustände zu überwinden, die zugrunde liegende Erkrankung zu behandeln und den Patienten wieder in einen stabilen Gesundheitszustand zu bringen.

### Palliativversorgung und Sterbebegleitung

Bei der Palliativversorgung steht die Symptomkontrolle im Vordergrund. Weitere Ziele sind eine Verbesserung der patientenzentrierten Kommunikation, ethische Beratung, Vorausverfügungen und Zielvereinbarungen. Angehörige berichten von größerer Zufriedenheit und weniger Stress, unter anderem weil sich die Kommunikation und die Beteiligung an der Patientenversorgung durch gemeinsame Entscheidungsfindung verbessern. Eine Verbesserung des ethischen Klimas durch die rechtzeitige Integration palliativmedizinischer Behandlungskonzepte kann zudem die moralische Belastung innerhalb des Teams verringern [27].

Bei Patienten auf der Intensivstation, für die eine Therapiebegrenzung beschlossen wurde, ist eine hochwertige palliative Versorgung unerlässlich. Die konsiliarische palliativmedizinische Betreuung in der Intensivmedizin hat in den letzten zwei Jahrzehnten zunehmend an Bedeutung gewonnen und spiegelt sich in einem konsultativen Modell wider [28]. Moderne Modelle aus der Intensivmedizin zielen zudem darauf ab, Palliativprinzipien und -interventionen in die tägliche Praxis des Intensivteams für alle Patienten zu integrieren (integratives Modell). Dies beinhaltet Schulungen zu Symptommanagement, Kommunikation und Entscheidungsfindung sowie die Entwicklung standardisierter Herangehensweisen für palliative Sedation, Extubationen und die Beendigung invasiver intensivmedizinischer Therapien wie Beatmung oder Nutzung von ECLS-Systemen. Frühzeitige palliativmedizinische Interventionen mit konsultativen oder integrativen Ansätzen verbessern die Symptomkontrolle und erhöhen die Übereinstimmung der Behandlung mit den Patientenwünschen. Die meisten Interventionen führen zu einer Verkürzung der Aufenthaltsdauer auf der Intensivstation und im Krankenhaus, ohne die Mortalität negativ zu beeinflussen [29, 24].

### Zeitlich begrenzter Therapieversuch auf der Intensivstation

Der TLT auf der Intensivstation dient als verbindliche Übereinkunft zwischen Behandlungsteam und Patient oder dessen juristischem Vertreter über ein Behandlungskonzept für einen definierten Zeitraum. Der TLT wird eingesetzt, um Unsicherheiten bezüglich der Prognose und des patientenzentrierten Behandlungsziels zu reduzieren. Er ermöglicht eine strukturierte Evaluation und Entscheidung über die Fortführung oder Beendigung intensivmedizinischer Maßnahmen basierend auf dem (mutmaßlichen) Patientenwillen. Durch klare Zielvereinbarungen und regelmäßige Reevaluation kann der TLT Überbehandlung vermeiden und eine patientenzentrierte Therapie fördern [25].

Der TLT kann als Alternative zu sofortigen Entscheidungen und als „bridge to decision“ bei Dissens dienen, um zusätzliche Zeit zu gewinnen und eine fundierte Entscheidung zu ermöglichen. Nachteile können allerdings auftreten, wenn zuvor definierte Ziele nicht eingehalten werden, was zu unnötigen Therapieverlängerungen führt und sowohl für den Patienten als auch für das Behandlungsteam belastend sein kann. Spätere Therapiezieländerungen werden dadurch erschwert. In solchen Fällen besteht die Gefahr, dass die Prognostizierung unsicher bleibt und moralischer Stress sowie Burn-out im Behandlungsteam verstärkt werden. Letztlich sollte man sich der Vor- und Nachteile dieser Methode bewusst sein und nicht im Rahmen einer „Futility-Situation“ agieren.

## Ressourcenallokation und Triage in der Intensivmedizin

In Zeiten knapper Ressourcen müssen ethisch vertretbare Triageprotokolle und Richtlinien zur fairen Verteilung angewendet werden. Zentrale Aspekte dabei sind

- Gerechtigkeit und Nichtdiskriminierung,
- Nutzenmaximierung,
- Transparenz und Rechenschaftspflicht,
- interdisziplinäre Entscheidungsfindung,
- Belohnung des instrumentellen Werts und
- Priorität für die am schwersten Betroffenen.

Ein effektives System, das diese Prinzipien vereint, ist das „complete lives system“. Es kombiniert verschiedene ethische Prinzipien und priorisiert jüngere Patienten, solche mit besserer Prognose sowie die Rettung der meisten Leben, um Diskriminierung und Manipulation zu vermeiden.

Gleichzeitig müssen Überversorgung und deren Ursachen adressiert werden. Regelmäßige Therapiezielevaluationen, interprofessionelle Kommunikation, Fortbildung und kritischer kollegialer Austausch im Team sind essenziell.

Therapie- und Triageentscheidungen bei limitierten Ressourcen sollten von einem Team getroffen werden, das nicht direkt in die Patientenversorgung involviert ist. Es sollten transparente Richtlinien übergeordneter Ebenen verwendet werden, um Interessenkonflikte und Voreingenommenheit zu vermeiden [14–19].

## Herausforderungen und zukünftige Entwicklungen in der Unterstützung von Grenzentscheidungen

Der Einsatz von künstlicher Intelligenz (KI) in der Intensivmedizin birgt erhebliche Chancen und Herausforderungen. KI kann die frühzeitige Erkennung und Prädiktion einer Sepsis ermöglichen, was eine schnellere Therapieeinleitung erlaubt [30]. Ebenso kann sie die mechanische Ventilation und Sedierung optimieren. Allerdings gibt es auch ethische und rechtliche Implikationen. Insbesondere müssen Haftungsfragen bei Fehlern oder Schäden durch KI-Systeme geklärt werden. Die Verantwortung könnte bei den Entwicklern, den Krankenhäusern oder den Ärzten liegen, die diese Systeme nutzen [31]. Bisher ist dieser Punkt jedoch ungeklärt.

Für den Einsatz von KI-Modellen, besonders von Black-Box-Systemen, ist essenziell, dass deren Entscheidungen nachvollziehbar und erklärbar sind, um eine angemessene klinische Anwendung und Überwachung zu gewährleisten. KI sollte Ärzte unterstützen und nicht deren Rolle ersetzen. Menschlicher Kontakt und Erfahrung bleiben für eine umfassende Patientenversorgung unersetzlich. Zudem muss die Gefahr von Verzerrungen in den Trainingsdaten, die zu einer ungerechten Behandlung bestimmter Patientengruppen führen können, dringend dargelegt werden.

## Resümee

Grenzentscheidungen in der Intensivmedizin erfordern eine strukturierte Vorgehensweise. Es ist wichtig, frühzeitig offene und ehrliche Gespräche mit den Patienten und deren Angehörigen über Prognose und Behandlungsmöglichkeiten zu führen. Regelmäßige multidisziplinäre Fallbesprechungen sollten genutzt werden, um alle relevanten Perspektiven einzubeziehen.

Die Autonomie des Patienten muss respektiert werden, indem Patientenverfügungen und Vorsorgevollmachten berücksichtigt werden. Entscheidungen sollten stets auf den ethischen Prinzipien der Patientenautonomie, Benefizienz, Nonmalefizienz und Gerechtigkeit basieren. Prognosemodelle und Scoresysteme sind hilfreiche Werkzeuge zur Unterstützung der Einschätzungen und sollten angewendet werden, um die nach der intensivmedizinischen Behandlung zu erwartende Lebensqualität des Patienten zu beurteilen und zu berücksichtigen. Die Rolle neuer Techniken aus dem Bereich des maschinellen Lernens zur Unterstützung dieser Entscheidungen bleibt ungeklärt, entsprechend finden solche Techniken bisher keine Anwendung.

Regelmäßige Reevaluationen und gegebenenfalls zeitlich begrenzte Therapieversuche helfen, die Therapieziele zu überprüfen und bei Bedarf anzupassen. Bei schwierigen Angehörigenkonstellationen oder komplexen ethischen Fragen können Ethikkonsultationen hilfreich sein. Alle Entscheidungsprozesse sind sorgfältig zu dokumentieren, eine transparente Kommunikation sollte gefördert werden. Ebenfalls wichtig ist, die kontinuierliche Fortbildung des medizinischen Personals in ethischen Fragestellungen und Kommunikationstechniken zu fördern. Diese strukturierte Vorgehensweise stellt sicher, dass Grenzentscheidungen in der Intensivmedizin zu einem ganzheitlichen und patientenorientierten Behandlungsansatz beitragen.

## Fazit für die Praxis

- Grenzentscheidungen in der Intensivmedizin erfordern eine strukturierte Vorgehensweise.
- Wichtig ist eine offene Kommunikation mit den Patienten und deren Angehörigen über Prognose und Behandlungsmöglichkeiten.
- Regelmäßige multidisziplinäre Fallbesprechungen beziehen alle relevanten Perspektiven ein.
- Entscheidungen sollten stets auf den ethischen Prinzipien der Patientenautonomie, Benefizienz, Nonmalefizienz und Gerechtigkeit basieren.
- Prognosemodelle und Scoresysteme sind hilfreich dabei, die nach der intensivmedizinischen Behandlung zu erwartende Lebensqualität einzuschätzen.
- Regelmäßige Reevaluationen und gegebenenfalls zeitlich begrenzte Therapieversuche helfen, die Therapieziele zu überprüfen und bei Bedarf anzupassen.
- Bei komplexen ethischen Fragen können Ethikkonsultationen hilfreich sein.

## Abkürzungen

APACHE: Acute Physiology And Chronic Health Evaluation
CFS: Clinical Frailty Scale
ECLS: „Extracorporeal life support“
KI: Künstliche Intelligenz
NEMS: Nine Equivalents of Nursing Manpower Use Score
SAPS: Simplified Acute Physiology Score
SF-36: Short-Form-Gesundheitsfragebogen mit 36 Items
SOFA: Sequential Organ Failure Assessment
TISS: Therapeutic Intervention Scoring System
TLT: „Time-limited trial“ (zeitlich begrenzter Therapieversuch)
